# Dynamic Interaction Between Mucosal Immunity and Microbiota Drives Nose and Pharynx Homeostasis of Common Carp (*Cyprinus carpio*) After SVCV Infection

**DOI:** 10.3389/fimmu.2021.769775

**Published:** 2021-11-04

**Authors:** Zheng-Ben Wu, Kai-Feng Meng, Li-Guo Ding, Sha Wu, Guang-Kun Han, Xue Zhai, Ru-Han Sun, Yong-yao Yu, Wei Ji, Zhen Xu

**Affiliations:** ^1^ Department of Aquatic Animal Medicine, College of Fisheries, Huazhong Agricultural University, Wuhan, China; ^2^ State Key Laboratory of Freshwater Ecology and Biotechnology, Institute of Hydrobiology, Chinese Academy of Sciences, Wuhan, China

**Keywords:** microbiota, mucosal immunity, common carp, nose, pharynx, spring viremia of carp virus (SVCV)

## Abstract

The crosstalk between the immune system and microbiota drives an amazingly complex mutualistic symbiosis. In mammals, the upper respiratory tract acts as a gateway for pathogen invasion, and the dynamic interaction between microbiota and mucosal immunity on its surface can effectively prevent disease development. However, the relationship between virus-mediated mucosal immune responses and microbes in lower vertebrates remains uncharacterized. In this study, we successfully constructed an infection model by intraperitoneally injecting common carp (*Cyprinus carpio*) with spring viremia of carp virus (SVCV). In addition to the detection of the SVCV in the nose and pharynx of common carp, we also identified obvious histopathological changes following viral infection. Moreover, numerous immune-related genes were significantly upregulated in the nose and pharynx at the peak of SVCV infection, after which the expression levels decreased to levels similar to those of the control group. Transcriptome sequencing results revealed that pathways associated with bacterial infection in the Toll-like receptor pathway and the Nod-like receptor pathway were activated in addition to the virus-related Rig-I-like receptor pathway after SVCV infection, suggesting that viral infection may be followed by opportunistic bacterial infection in these mucosal tissues. Using 16S rRNA gene sequencing, we further identified an upward trend in pathogenic bacteria on the mucosal surface of the nose and pharynx 4 days after SVCV infection, after which these tissues eventually reached new homeostasis. Taken together, our results suggest that the dynamic interaction between mucosal immunity and microbiota promotes the host to a new ecological state.

## Introduction

The mucosa of vertebrates is a particularly dynamic environment in which trillions of commensal microorganisms exist, known as the microbiota, which plays an important role in many biological functions including growth enhancement, nutrition, development, and metabolism (1, 2). Recently, the microbiota has been found to be closely related to the host’s immune system, as it induces the occurrence of immune responses and enhances immune susceptibility (3). In response, the host mucosal immune system has evolved multiple means to maintain its symbiotic relationship with the microbiota (4). Unlike invertebrates, teleost fish have evolved both innate and adaptive immunity due to evolutionary pressures and can therefore protect themselves against pathogens residing in the aquatic environment and maintain mucosal microbiota homeostasis (2, 5). Given the uniqueness and complexity of the living environment of teleost, the mucosal tissues on the surface of these organisms are the first to contact the pathogen and are therefore the first line of defense against pathogen invasion. So far, six different mucosa-associated lymphoid tissues (MALTs) have been identified in teleost fish, including gut-associated lymphoid tissue, gill-associated lymphoid tissue, skin-associated lymphoid tissue, buccal mucosa-associated lymphoid tissue, nasal-associated lymphoid tissue, and pharyngeal mucosa-associated lymphoid tissue, all of which lack the organized MALTs (O-MALTs) such as Peyer’s patches and tonsils but contain diffuse MALTs (D-MALTs) (5). Notably, unlike the mammalian nasopharynx, the nose and pharynx of teleost are two separate compartments that are not related due to the lack of a choana (6, 7). However, as the olfactory organ of fish, the nose is in direct and constant contact with water and air. Furthermore, the pharynx is located at the junction of the respiratory and digestive tract, which is also in constant contact with water and food. Additionally, the nose and pharynx exhibit strong adaptive immune response, with increased total secreted IgT and parasite-specific IgT, as well as local proliferation of IgT^+^ B cells in rainbow trout when invaded by *Ichthyophthirius multifiliis* (8, 9). It is worth noting that the innate immune response of the nasal and pharyngeal mucosa also plays an important role in defending the host against viral infection. The proinflammatory cytokines IL1β and IL8 were significantly upregulated in rainbow trout after IHNV infection, and antimicrobial immune responses were also detected in the mucosal tissues (10–13). Previous studies demonstrated that both the nasal and pharyngeal mucosa of teleost were involved in immune responses against pathogen invasion. However, secondary bacterial infection and the systematic changes of the immune pathways in the nasal and pharyngeal mucosa after virus infection remain uncharacterized.

Abundant microbes are found to colonize the upper respiratory tract, especially the nasopharyngeal cavity, which has been studied extensively in mammals (14). However, far few studies have focused on the microbial diversity of teleost nasal and pharyngeal mucosa. So far, nasal and pharyngeal mucosa commensal bacteria have only been characterized in rainbow trout, among which *Proteobacteria*, *Firmicutes*, *Bacteroidetes*, and *Actinobacteria* account for more than 80% of the total phylum-level diversity (10, 15). However, the harmonious relationship between the host and the microbiota can be disrupted by pathogen invasion, which leads to microbiota dysbiosis. Our previous study demonstrated that SVCV infection can disrupt the microbial homeostasis of both external (buccal mucosa, gills, and skin) and internal (gut) mucosal tissues in common carp, resulting in the proliferation of opportunistic pathogens coupled with a decrease in beneficial bacteria (16). Additionally, the host’s immune system also responds to microbial imbalances. For example, in the gill tissue of rainbow trout, sIgT coated both beneficial bacteria that may produce short-chain fatty acids (SCFAs) as well as potentially pathogenic bacteria, whereas an increase in IgM-coated microbiota was found after 3–6 weeks of IgT depletion, suggesting a possible IgM compensation response (17). Moreover, the antimicrobial peptides (AMPs), lectins, and mucins secreted by teleost are upregulated in response to pathogens, thus activating innate immune responses and also effectively insulating bacteria from mucosal epithelial cells and forming a chemical barrier (18–20). Therefore, we hypothesized that, in addition to effectively eliminating pathogens in the teleost nasal and pharyngeal mucosa, the immune response also enables the microbiota on the mucosal surface to achieve homeostasis.

The SVCV, which belongs to the genus *Vesiculovirus* of the *Rhabdoviridae* family, is an aquatic pathogen responsible for major disease outbreaks and large-scale mortality in cyprinids, especially common carp (*Cyprinus carpio*) (21). In this study, we successfully constructed an infection model where a less lethal concentration of the SVCV was administered to common carp *via* injection, after which the virus could successfully migrate to the nasal and pharyngeal mucosal tissues. Furthermore, viral infection led to a strong immune response in the nose and the pharynx. Transcriptome analysis and 16S rRNA sequencing further characterized the immune response and microbiota changes in the nasal and pharyngeal mucosa of common carp infected with the SVCV, as well as their dynamic interplay, suggesting the invasion of opportunistic pathogens and the possibility of secondary bacterial infection in these mucosal tissues after viral infection. Finally, with the decrease in the viral load, a new microbiota-immune homeostasis formed.

## Materials and Methods

### Fish Maintenance

The 5-month-old common carp (150-tailed) with a body weight of 10–15 g were purchased from an aquaculture farm in Chongqing, China, and then were transported to the fish-breeding base of Huazhong Agricultural University (HZAU). All the common carp were kept in an indoor circulating water tank with freshwater at 18°C. Fish were acclimatized for at least 14 days and fed with commercial carp pellets at a rate of 0.5%–1% body weight twice a day (9:00 a.m. and 4:00 p.m.) during the whole experimental period. The feeding was terminated at 48 h prior to sacrifice both in control and infected groups. Before the infection experiment, the fish were acclimatized to the water temperature by changing the water temperature from 18 to 12°C by 2°C per day for the SVCV infection.

### Virus Enrichment, Collection, and Infection

The cyprinus carpio epithelioma papillosum cyprini (EPC) cell line was maintained in a minimum Eagle’s medium (MEM) supplemented with 10% fetal bovine serum (FBS) and 1% penicillin-streptomycin solution, and cultured in an incubator at 26°C with 5% CO_2_. The SVCV was inoculated onto the EPC, and the presence of cytopathic effects (CPEs) was checked every day through microscopy. When CPEs were apparent in ∼80% of the monolayer, the mixture of cells and fluid were harvested, and then freeze-thawed three times and centrifuged at 1,000 *g* for 10 min at 4°C to collect the supernatant. Subsequently, the supernatant was diluted proportionally (10^-1^ ~ 10^-12^) and inoculated into 96-well cell culture plates with 90% monolayer EPC cells at the bottom, and CPEs were observed every 24 h for 7 days to calculate the TCID50 of the SVCV. Then the dose of the SVCV was adjusted to 1 × 10^7^ pfu ml^−1^ in MEM and stored at −80°C until use. The common carp were infected with the SVCV by intraperitoneal injection as described in a previous study (16). Briefly, the fish were anesthetized with sodium mesylate (MS-222) at a final concentration of 40 μg/ml and then injected with 100 μl MEM containing the SVCV. As the control group, the fish were also treated by intraperitoneal injection of 100 μl MEM collected from uninfected cells. Tissue samples including the nose, pharynx, blood, and spleen of common carp were collected from six individuals at 1, 4, 7, 14, and 28 days post infection (dpi) of the SVCV. Similarly, the same samples from six individuals of the control group were collected.

### Plaque Assay of SVCV

The nose and pharynx from control and infection groups were obtained and placed in clean petri dishes to remove muscle tissues. Thereafter, the tissues were cut into small pieces (~ 0.1 cm^2^) and agitated continuously in cold PBS (pH 7.2) for 3 min at 4°C. The tissue pieces were mechanically disaggregated on a 100-μm cell strainer, and the cell suspension was collected. All cell suspension was then freeze-thawed three times to break the cells and release the virus after adding 5% FBS, which can prevent virus inactivation. The cell suspension was centrifuged for 5 min at 400 g once and 10,000 *g* thrice at 4°C to remove large cell debris, and then the supernatant was filtered with a 0.45-μm membrane filter to remove bacteria in the mucosal tissue. The resulting liquid was added to a 6-well cell culture plate covered with 90% monolayer EPC cells at the bottom and maintained in 1.5 ml MEM. When CPEs were clearly apparent, the cells were fixed with 4% (v/v) neutral paraformaldehyde buffer for at least 3 h, then washed carefully with PBS (pH 7.2), and stained with crystal violet for 15 min. The results of plaque assay were examined under a light microscope (Phenix) and imaged on the MShot image analysis system.

### RNA Isolation and Quantitative Real-Time PCR (qRT-PCR) Analysis

Total RNA was extracted from various tissues using a TRIzol Reagent (Invitrogen, USA) according to the manufacturer’s protocol. The concentration of extracted RNA was determined by spectrophotometry (Nanodrop ND1000, LabTech), and the integrity of the RNA was determined by 1% agarose gel electrophoresis (Agilent Bioanalyser, 2100). To normalize gene expression levels for each sample, equivalent amounts of total RNA (1,000 ng) were used for cDNA synthesis with the SuperScript first-strand synthesis system in a 20-μl reaction volume. The synthesized cDNA was diluted three times and then used as a template for qRT-PCR analysis. The qRT-PCR was performed by the qTOWER3G PCR system (Analytik Jena AG, Germany) using the EvaGreen 2 × qPCR Master mix (YEASEN, China) as the following conditions: 95°C for 5 min, followed by 40 cycles at 95°C for 10 s and at 58°C for 30 s. The qRT-PCR products of the SVCV were verified by 2% agarose gel electrophoresis. Ct values determined for each sample were normalized against the values of 40S, and the change in transcription of genes was calculated as relative fold expression by the methods of 2^-ΔΔCt^. Primers used for qRT-PCR are listed in **Supplementary Table 1**.

### Standard Curve for SVCV

The method of making the standard curve has been described previously (16). Briefly, the PCR products of the SVCV were ligated into pMD 19-T vectors and then transformed into the competent cells of *Escherichia coli* DH5α. Plasmid DNA was isolated from an overnight selective culture using a HiPure Plasmid Micro Kit (OMEGA). The recombinant plasmids were diluted 10 times continuously (a total of 7 gradients of 1.27 × 10^8^ copies/ml ~1.27 × 10^2^ copies/ml) and used as the positive standard template. Then, Ct values and corresponding copy numbers were used to make a standard curve of the SVCV, which is shown in **Supplementary Figure 1**.

### Histology and Light Microscopy Studies

For histological and pathological studies, the nose and pharynx of common carp were taken directly from control and infected fish and then immediately fixed with 4% (v/v) neutral paraformaldehyde buffer for at least 24 h. Then, tissues were dehydrated in a graded ethanol series, washed with xylene, embedded in paraffin, and sectioned into 5-μm pieces. Thereafter, the paraffin sections were stained with hematoxylin and eosin (H&E) or alcian blue (AB) as described previously (8). Images were acquired in a microscope (Olympus) using the Axiovision software, and then the thickness of lamina propria and epidermis was measured. The number of mucous cells was counted by Photoshop (version 22.5). The parameters of each image were measured by three different researchers and averaged to reduce random errors.

### RNA-Seq Library Construction, Sequencing, and Data Analyses

The nose and pharynx samples of the control group and the SVCV infection group of 4 and 28 dpi were sent to Seqhealth Technology Co., Ltd. (Wuhan, China). Briefly, total RNA was extracted using a TRIzol reagent (Invitrogen, USA) and was used for stranded RNA sequencing library preparation using a KCTM Stranded mRNA Library Prep Kit for Illumina^®^ following the manufacturer’s instruction. PCR products corresponding to 200–500 bps were enriched, quantified, and finally sequenced on a Hiseq X 10 sequencer (Illumina). Reads were mapped to the *Cyprinus carpio* genome using STAR (version 2.5.3a) with default parameters (22). The mapped reads were analyzed by feature Counts (Subread-1.5.1; Bioconductor) (23). Differential expression genes were estimated by the edgeR package (version 3.12.1) (24). The genes with low expression (CPM (counter-per-million) < 1 in three or more samples) were excluded from downstream analysis. The resulting genes were considered as differentially expressed genes (DEGs) if FDR ≤ 0.05 and |log2 (fold-change) | ≥ 1. For further analysis of the DEGs, we carried out a Kyoto encyclopedia of genes and genomes (KEGG) enrichment using KOBAS (version: 2.1.1) (25) to identify the immune-related pathways that were significantly enriched following viral infection.

### Bacterial 16S rRNA Sequencing and Data Analyses

Same samples as in RNA-Seq were sent to Shanghai Personal Biotechnology Co., Ltd (Shanghai, China), where the total genomic DNA samples were extracted using the OMEGA Soil DNA Kit (M5635-02) (Omega Bio-Tek, Norcross, GA, USA), following the manufacturer’s instructions, and the quantity and quality of extracted DNA were measured using a NanoDrop NC2000 spectrophotometer (Thermo Fisher Scientific Inc) and agarose gel electrophoresis, respectively. The V3-V4 region of bacterial 16S rRNA was amplified by PCR using the forward primer 338F (5’-ACTCCTACGGGAGGCAGCA-3’) and the reverse primer 806R (5’-GGACTACHVGGGTWTCTAAT-3’). Sample-specific 7-bp barcodes were incorporated into the primers for multiplex sequencing. After completion of PCR, the amplicons were purified with Vazyme VAHTSTM DNA Clean Beads (Vazyme, Nanjing, China) and quantified using the Quant-iT PicoGreen dsDNA Assay Kit (Invitrogen, Carlsbad, CA, USA). And then, amplicons were pooled in equal amounts, and pair-end 2×250 bp sequencing was performed using the Illlumina MiSeq platform with MiSeq Reagent Kit v3. Raw sequence data were demultiplexed using the demux plugin and were quality filtered, denoised, merged, and chimera removed using the DADA2 plugin. Sequence data analyses were mainly performed using QIIME2 (26) and R packages (v3.2.0). Briefly, the taxonomy compositions and abundances were visualized using MEGAN (27) and GraPhlAn (28). Beta diversity analysis was performed to investigate the structural variation of microbial communities across samples using nonmetric multidimensional scaling (NMDS) hierarchical clustering (29). LEfSe (Linear discriminant analysis effect size) was performed to detect differentially abundant taxa across groups using the default parameters (30). Network was visualized using the R package igraph and ggraph.

### Statistics

An unpaired Student’s *t*-test (Prism version 6.01; GraphPad) was used for analysis of differences between groups. Statistical significance was checked by setting the alpha at 0.05 for all analyses.

### Availability of Data

The NCBI Sequence Read Archive (SRA) accession numbers for the transcriptome and microbiome data reported in this manuscript are PRJNA761005 (https://www.ncbi.nlm.nih.gov/bioproject/PRJNA761005/) and PRJNA760965 (https://www.ncbi.nlm.nih.gov/bioproject/PRJNA760965/), respectively.

## Results

### Successful Construction of the SVCV Infection Model in Common Carp

**Figure 1:**
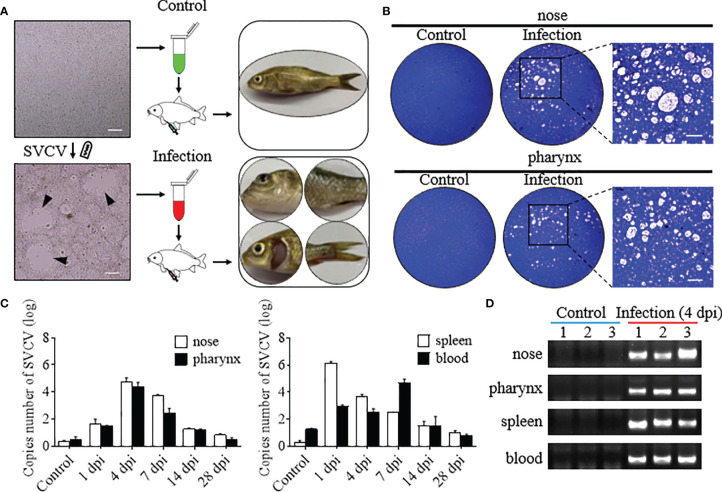
Successful construction of the model of common carp infected with the SVCV. **(A)** The flowchart represented the propagation of the SVCV (left), the intraperitoneal injection into common carp (middle), and the characterization after SVCV infection (right), respectively. The top and bottom were the treatment of the control and infected groups, respectively. Black triangles indicate plaque. Scale bar, 100 μm. **(B)** Plaque assays detected SVCV virion with infectious activity in the nose and pharynx of the control and infected groups, and the enlarged images showed significant cytopathic effect (CPE) in the infected group. Scale bar, 50 μm (*n* = 3 fish per group). **(C)** The number of SVCV copies was detected in the nose, pharynx, spleen, and blood in the control and infected groups at 1, 4, 7, 14, and 28 dpi (*n* = 6 fish per group). **(D)** The SVCV was amplified by qPCR in the nose, pharynx, spleen, and blood of the control and infected (4 dpi) groups and detected by agarose gel electrophoresis (*n* = 3 fish per group).

To evaluate the interaction between the immune response and the microbiota in the mucosal tissue, we constructed the infection model with SVCV. At 3 dpi, clear clinical signs were observed in infected common carp, including exophthalmos, pale gills, loss of scales, and caudal fin bleeding (**Figure 1A**). Furthermore, homogenates of nose and pharynx tissues from control and infected fish were filtered, and reinfected EPC cells, obvious CPEs, and a certain number of plaques were observed in infected groups EPC cells, whereas no such CPEs and plaques were observed in control groups of EPC cells (**Figure 1B**). In addition, to further determine the path of virus invasion and the changes of virus load over time, we measured the number of SVCV copies in the nose, pharynx, spleen, and blood. Interestingly, the highest SVCV copies were detected in the nose and pharynx at 4 dpi (~1.1×10^5^ and ~6×10^4^, respectively). As expected, high copies of the SVCV were detected in the spleen and blood beginning at 1 dpi, with the highest copies at 1 dpi (~1.6×10^6^) in the spleen and at 7 dpi (~9.5×10^4^) in the blood (**Figure 1C**). And the results of agarose gel electrophoresis further verified the virus load intuitively, in which the positive bands of the SVCV could be detected in the nose, pharynx, spleen, and blood at 4 dpi but not in the control fish (**Figure 1D**). Overall, these results signified that the SVCV infection model of common carp was successfully constructed, and the SVCV can invade into the mucosal tissues of the nose and pharynx and gradually decreases over time.

### SVCV Infection Induced Morphological Changes in Nose and Pharynx of Common Carp

**Figure 2:**
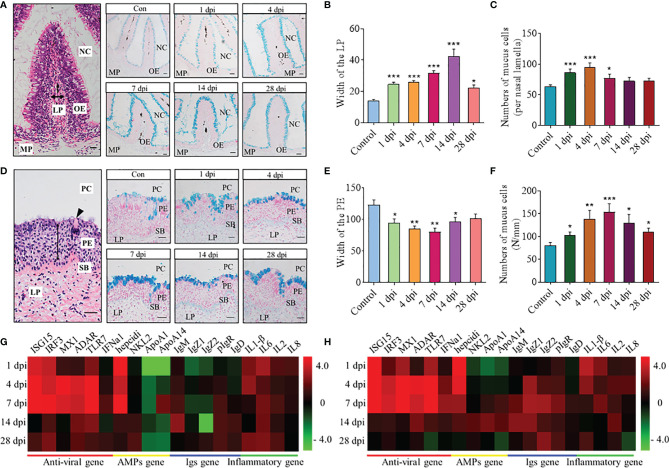
Histopathological changes and expression of immune-related genes in the nose and pharynx of common carp after SVCV infection. **(A, D)** Histological examination (H&E and AB staining) of olfactory organs **(A)** and pharynx **(D)** from control fish (Con) and SVCV-infected fish at 1, 4, 7, 14, and 28 dpi. Two-way arrowhead indicates LP and PE, and black triangle indicates taste bud. Scale bar, 20 μm. **(B, E)** The width of LP at the tip region (100 μm from the lamellar tip) of the nasal villi **(B)** and PE of the pharynx **(E)** in control fish and SVCV-infected fish at 1, 4, 7, 14, and 28 dpi counted from A (*n* = 6 fish per group). **(C, F)** The number of mucus cells per nasal lamella **(C)** and per millimeter in the PE **(F)** in control fish and SVCV-infected fish at 1, 4, 7, 14, and 28 dpi counted from A (*n* = 6 fish per group). **(G, H)** Heatmap illustrates results from qRT-PCR of mRNAs for selected immune-related genes (antiviral gene, AMP gene, Igs gene, and inflammatory gene) in nose **(G)** and pharynx **(H)** of SVCV-infected fish *versus* control fish at 1, 4, 7, 14, and 28 dpi (*n* = 6 fish per group). Data are expressed as mean cycle threshold (Ct) values ± SEM. NC, nasal cavity; OE, olfactory epithelium; MP, muscularis propria; LP, lamina propria; PC, pharyngeal cavity; PE, pharyngeal epithelium; SB, stratum basale. *p < 0.05, **p < 0.01, ***p < 0.001 (unpaired Student’s t test).

To understand the histological organization of nasal and pharyngeal mucosa of common carp, their paraffin sections were stained with H&E. The olfactory organs are composed of nasal cavity (NC), olfactory epithelium (OE), lamina propria (LP), and muscularis propria (MP) (**Figure 2A**), and the pharyngeal mucosa is composed of pharyngeal cavity (PC), pharyngeal epithelium (PE), stratum basale (SB), and lamina propria (LP) (**Figure 2D**). By AB staining, the pathological changes and mucous cells in nasal and pharyngeal mucosa after infection with the SVCV could be observed obviously over time (**Figures 2A, D** **)**. In olfactory organs, the width of the LP at the tip region (100 μm from the lamellar tip) gradually enlarged from 1 to 14 dpi and showed a trend of recovery at 28 dpi, which all increased significantly compared with the control fish (**Figure 2B**). Moreover, the width of the OE tends to be thinner at 4 and 7 dpi, and the number of mucus cells in the OE was increased significantly at 1, 4, and 7 dpi (**Figure 2C** and **Supplementary Figure S2**). Intriguingly, similar to the changes in the OE, the width of the PE was showed to be significantly thinner than the control fish at 1, 4, 7, and 14 dpi, and the number of mucus cells in the PE was significantly increased from 1 to 28 dpi (**Figures 2E, F** **)**. All these results indicated that SVCV infection can destroy the structure of nasal and pharyngeal mucosa to a certain extent and the potential role of the OE and the PE in defense of virus invasion.

### SVCV Infection Induced Immune Gene Expression in Nose and Pharynx of Common Carp

Using quantitative real-time PCR (qRT-PCR), we measured the relative expression levels of 19 immune-related genes in the nose and pharynx of common carp at 1, 4, 7, 14, and 28 dpi (**Figures 2G, H** **)**, including 6 antiviral genes (ISG15, IRF3, MX1, ADAR, TLR7, and IFNa1), 4 antimicrobial peptide (AMP) genes (hepcidin, NKL2, APOA1, and APOA14), 5 immunoglobulin (Ig) genes (IgM, IgZ1, IgZ2, IgD, and pIgR), and 4 inflammatory genes (IL1β, IL6, IL2, and IL8). Importantly, our results showed that the gene expression profiles in the nose and pharynx were similar, with the mRNA expression of antiviral genes and inflammatory genes significantly upregulated at 4 and 7 dpi. Moreover, the expression of hepcidin was also upregulated significantly at 1, 4, and 7 dpi, and the higher expression level of IgM, IgD, and pIgR was expressed at 14 dpi. However, there were also differences in the gene expression between the nose and pharynx, such as the fact that the expression of APOA1 and APOA14 was downregulated in the nose (~0.57 and 0.41-fold, respectively) but upregulated in the pharynx (~3.75 and 2.8-fold, respectively) at 14 dpi, and both of them were shown to display bactericidal and/or bacteriostatic activity. Notably, compared with 1, 4, 7, and 14 dpi, the expression of all genes decreased to some extent at 28 dpi and tended to the control level.

### Kinetics of Immune Response in Nose and Pharynx After SVCV Infection

To further study the dynamics of the immune response in the nose and pharynx of common carp after SVCV infection, RNA-seq was implemented and samples of two time points, 4 and 28 dpi, were selected for comparative analysis with the control (Con) group in the present study. After sequencing on the Illumina platform, we have gained a comprehensive transcriptomic profile from the nose and pharynx of common carp. By filtering the sequences with thresholds and removing the repeats, they were compared to the reference genome of common carp. Finally, we obtained 122,080,476, 136,130,076, and 130,227,455 total reads in the nose of control, 4 dpi, and 28 dpi groups, respectively, and 149,921,168, 157,835,249, and 138,197,558 total reads in the pharynx of control, 4 dpi, and 28 dpi groups, respectively. Afterward, unique mapped reads were further filtered, and only more than 10 reads in three or more individual libraries were used for analyses of differential expression genes (DEGs).

**Figure 3:**
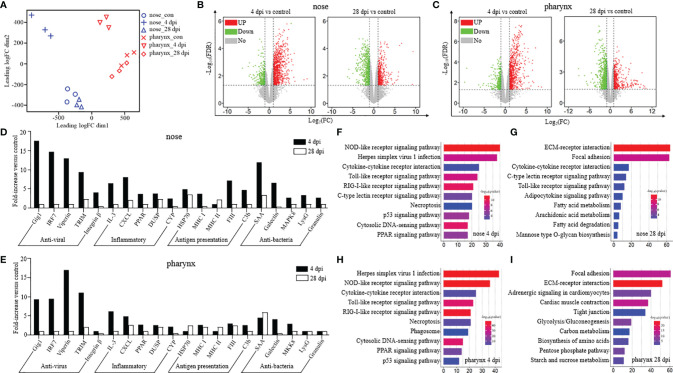
Kinetics of the immune response was analyzed from the perspective of transcriptome in the nose and pharynx of common carp after being infected with the SVCV (*n* = 3 fish per group). **(A)** PCA (principal component analysis) cluster plots of gene expression levels in different samples. The horizontal axis represents the first ranking principal component dimension, and the vertical axis represents the second ranking principal component dimension. Blue and red indicate nose and pharynx, respectively, and different shapes indicate different groups. **(B, C)** Volcano plot displayed the DEG distribution in the nose **(B)** and pharynx **(C)** of common carp at 4 dpi (left) and 28 dpi (right) compared with the control group. Red spots, expression fold change of >2 and FDR of <0.05. Green spots, expression fold change of <2 and FDR of <0.05. Gray spots, no difference in expression. The horizontal axis represents -log_10_(FDR), and the vertical axis represents log_2_(FC). **(D, E)** Representative immune-related genes (antiviral, antigen presentation, inflammatory, and antibacteria genes) in nose **(D)** and pharynx **(E)** modulated by SVCV infection on day 4 or 28. Data are expressed as mean fold increase in expression. **(F–I)** KEGG pathways were significantly altered in nose **(F, G)** and pharynx **(H, I)** of common carp at 4 and 28 dpi *versus* control fish revealed by RNA-Seq studies. Fold change differences between control and SVCV-infected groups were calculated using cutoff of twofold.

Prior to DEG analysis, principal component analysis (PCA) was performed on the filtered genes to examine congruency among biological replicates and visualize the distribution of various groups in the nose and pharynx (**Figure 3A**). As shown, the Con and 28 dpi groups clustered more closely compared to the 4 dpi group in both the nose and pharynx. Moreover, the PCA plot showed that the groups of the nose were separated from the groups of the pharynx, and a high level of consistency was present in the biological replicates of the same sites of tissues. Next, the expression pattern of DEGs was analyzed in the Con/4 dpi and Con/28 dpi groups of the nose and pharynx using the volcano plots, respectively (**Figures 3B, C** **)**. Notably, 1,424 genes in the 4 dpi group and 1,245 genes in the 28 dpi group were differentially regulated in the nose following SVCV infection, in which 1,059 genes and 563 genes were upregulated, and 365 genes and 682 genes were downregulated at 4 and 28 dpi, respectively. Interestingly, the same trends of DEGs were found in the pharynx, which showed that 1,306 genes in the 4 dpi group and 1,700 genes in the 28 dpi group were differentially regulated after infection. Of these, 910 and 683 genes were upregulated, and 396 and 1,017 genes were downregulated in the pharynx at 4 and 28 dpi, respectively. Based on the up- and downregulated genes, we selected 20 representative immune-related genes and analyzed their expression level changes at 4 and 28 dpi in both the nose and pharynx after SVCV infection, including antiviral genes (grass carp reovirus-induced gene 1, Gig1; interferon regulatory factor 7, IRF7; Viperin; Tripartite motif, TRIM; and Integrin-β), inflammatory genes (interleukin 3, IL3; chemokine (C-X-C motif) ligand, CXCL; peroxisome proliferator-activated receptor, PPAR; dual-specificity phosphatases, DUSP; and cytochrome P450, CYP), antigen presentation-related genes (heat shock protein 70, HSP70; major histocompatibility complex I, MHCI; major histocompatibility complex II, MHCII; coagulation factor III, FIII; and complement 3b, C3b), and antibacteria genes (serum amyloid A protein, SAA; galectin; mitogen-activated protein kinase 8, MAPK8; lysozyme g, LysG; and granulin) (**Figures 3D, E** **)**. The same expression pattern of immune-related genes was found in the nose and pharynx, which indicated that similar immune response was induced to resist to virus invasion, but there existed a difference, with the expression levels of antigen presentation-related genes generally slightly higher in the nose than in the pharynx. Interestingly, more antibacteria genes were also upregulated at 4 dpi in the nose when compared to that of the pharynx, in which only three genes (SAA, galectin, and MAPK8) were upregulated at 4 dpi, suggesting that viral invasion could lead to secondary bacterial infection. To validate the accuracy of RNA-Seq results, seven genes were randomly selected to detect their expression levels by qRT-PCR (**Supplementary Figures 4A, B** **)**. As shown, the significant correlation of the expression was found by qRT-PCR and RNA-Seq, indicating the reliability of transcriptome data.

### Activation of Pattern Recognition Receptors (PRRs) Pathways in Nose and Pharynx After SVCV Infection

**Figure 4:**
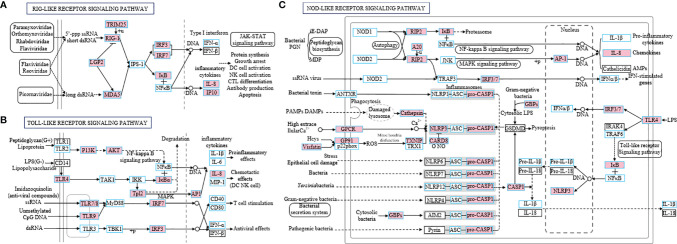
Three specific families of pattern recognition receptors, RIG-I-like receptor signaling pathway **(A)**, Toll-like receptor signaling pathway **(B)**, and NOD-like receptor signaling pathway **(C)**, were responsible for detecting various pathogens and generating innate immune responses. These pathways were simplified and modified from KEGG. The genes boxed with blue border in the pathways were identified in common carp. Genes marked in pink represent DEGs in RNA-seq.

Based on the transcriptome data, we further performed the KEGG pathway enrichment analysis to investigate the functions of the identified DEGs among the three groups. The results showed that the pathways associated with immune response were enriched in the differentially expressed set of genes in the nose and pharynx at 4 dpi, sharing the same top 5 pathways, including the NOD-like receptor (NLR) signaling pathway, Herpes simplex virus 1 infection, cytokine–cytokine receptor interaction, the Toll-like receptor (TLR) signaling pathway, and the RIG-I-like (RLR) receptor signaling pathway (**Figures 3F, H** **)**. However, at 28 dpi, the DEGs of the nose and pharynx were most enriched in signal molecules and metabolism pathways, such as the ECM–receptor interaction pathway and the Focal adhesion signaling pathway, which were identified as important processes or signaling pathways (**Figures 3G, I** **)**. Moreover, three crucial signaling pathways responsible for recognizing various pathogens and generating innate immune responses were analyzed, including the RLR signaling pathway (**Figure 4A**), the TLR signaling pathway (**Figure 4B**), and the NLR signaling pathway (**Figure 4C**). Meanwhile, we found that the key node genes RIG-1 and MDA5 in the RLR signaling pathway were significantly upregulated both in the nose and pharynx (**Supplementary Figure 3A**), which play an important role in the detection of the SVCV. In addition, TLR7/8 and TLR9 related to virus recognition in the TLR signaling pathway were also upregulated (**Supplementary Figure 3B**). PI3K, AKT, TLR4, and IκBα node genes related to bacteria recognition involved in the TLR signaling pathway were activated. Similar to the results in the TLR signaling pathway, we also found the activation of the bacterial recognition genes involved in the NLR signaling pathway, among which RIP2, A20, IκB, and AP-1 node genes were involved in recognition of bacterial peptidoglycan (PGN), TLR4 involved in lipopolysaccharide (LPS) recognition, and GBPs involved in cytosolic bacteria recognition. All these genes were significantly differentially expressed at 4 dpi in the nose and pharynx (**Supplementary Figure 3C**). These results suggest that SVCV infection may induce secondary bacterial infection of the mucosal surfaces of the nose and pharynx in common carp.

### Microbial Dysbiosis Occurred in Nose and Pharynx After SVCV Infection

**Figure 5:**
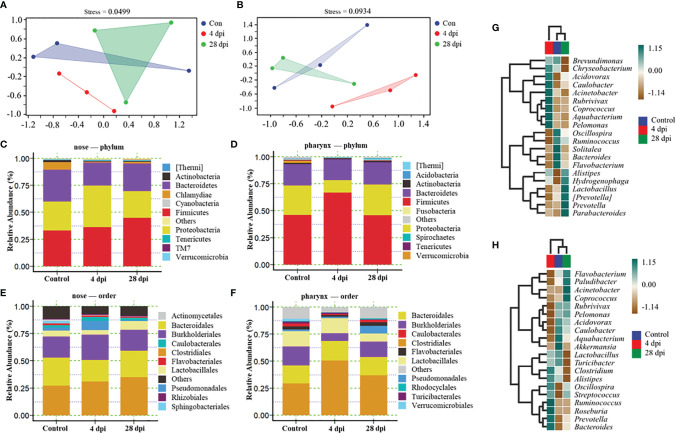
Changes in microbiota composition in the nose and pharynx from control and SVCV-infected common carp (*n* = 3 fish per group). **(A, B)** NMDS analysis showed the community differences among the samples in the nose **(A)** and pharynx **(B)** of common carp. Blue indicated the control group, red indicated the 4 dpi group, and green indicated the 28 dpi group. **(C, D)** Composition and relative abundance of the top 10 dominant bacterial taxa in the nose **(C)** and pharynx **(D)** of common carp in control fish and SVCV-infected at 4 and 28 dpi fish at the phylum level. **(E, F)** Composition and relative abundance of the top 10 dominant bacterial taxa in the nose **(E)** and pharynx **(F)** of common carp in control fish and SVCV-infected at 4 and 28 dpi fish at the order level. **(G, H)** Heatmap further compared the composition of the top 20 genera with average abundance in different groups in the nose **(G)** and pharynx **(H)** of common carp. Blue indicated the control group, red indicated the 4 dpi group, and green indicated the 28 dpi group.

The nose and pharynx are important mucosal tissues and colonized by a large number of microorganisms. In order to verify the changes of microbial composition and distribution in the nose and pharynx of common carp after SVCV infection, we performed 16S rRNA sequencing with the Illumina miseq platform. After the sequences were filtered and spliced, we picked 8,959 efficient operational taxonomic units (OTU) in the nose, with 2,726 OTU in the control group, 3,703 OTU in the 4 dpi group, and 3,896 OTU in the 28 dpi group, and 9,635 efficient OTU in the pharynx, with 3,406 OTU in the control group, 3,339 OTU in the 4 dpi group, and 4,120 OTU in the 28 dpi group. Nonmetric multidimensional scaling (NMDS) analysis showed that samples of the nose at 4 dpi were tightly clustered and separated from those of the control and 28 dpi groups, which were scattered and had overlapped with each other, and the similar results were found in the pharynx, indicating that the SVCV may cause microbial dysbiosis 4 days after infection (**Figures 5A, B** **)**. To further analyze the composition of microbiota in the nose and pharynx of common carp, we classified the phylum, class, order, family, and genus of microbial sequences from the control and infected fish. The results showed that a total of top 10 phyla were observed both in the nose and pharynx, with *Firmicutes*, *Proteobacteria*, and *Bacteriodetes* being the most dominant phyla between infected and control groups (**Figures 5C, D** **)**. Concretely, the abundance of *Firmicutes* present in the nose increased from 33% in the control group to 36% and 44% in the 4 and 28 dpi groups, respectively. Importantly, the abundance of *Proteobacteria* in the nose increased from 27% in the control group to 38% in the 4 dpi group but dropped to 24% in the 28 dpi experimental group. By contrast, a decreased abundance of *Bacteriodetes* was detected (21%) at 4 dpi in the nose and then returned to 25% at 28 dpi, similar to the control group (29%) (**Figure 5C** **)**. In the pharynx, the abundance of *Firmicutes* increased (67%) significantly at 4 dpi and returned to similar abundance (46%) as the control group at 28 dpi. However, *Proteobacteria* decreased significantly to 11% at 4 dpi and then increased to 28% at 28 dpi, similar to that of the control group. Different from that in the nose, the abundance of *Bacteriodetes* in the pharynx remained at 19% throughout the experimental period (**Figure 5D** **)**. At the order level, although the changes in the microbial composition of the nose were moderate except *Clostridiales*, we observed clearly significant microbial changes in the pharynx, including pathogenic bacteria *Burkholderiales* and *Pseudomonadales*, together with beneficial bacteria *Clostridiales* and *Lactobacillales*, which further confirmed the diversity of microbial changes between the nose and pharynx in teleost due to geographical differences (**Figures 5E, F** **)**. Details about the changes of the microbial community at the genus level were shown with heatmaps including the top 20 bacteria from control and infected groups (**Figures 5G, H** **)**. Interestingly, the abundance of *Acidovorax*, *Caulobacter*, *Rubrivivax*, and *Pelomonas* increased in the nose but decreased in the pharynx at 4 dpi. In addition, we observed the abundance of *Acinetobacter* and *Coprococcus* increased significantly in the nose of common carp at 4 dpi, but a similar increase occurred in the pharynx at 28 dpi. On the contrary, the abundance of *Alistipes* and *Prevotella* was significantly increased in the pharynx at 4 dpi and then ascended in the nasal at 28 dpi.

### Significantly Changed Microbial Community in Nose and Pharynx After SVCV Infection

**Figure 6:**
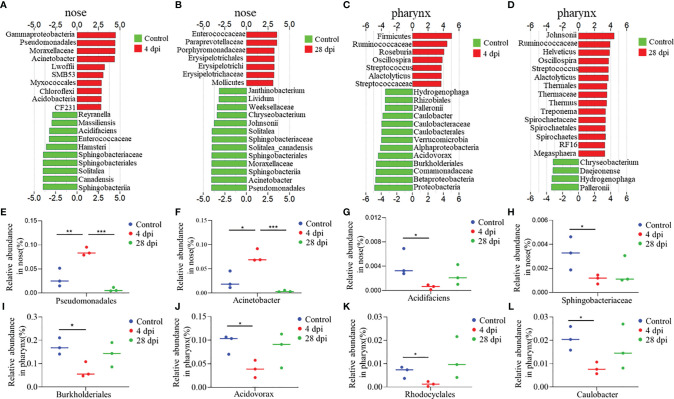
Description of biomarkers was significantly changed between the control and infection groups at 4 or 28 dpi in common carp (*n* = 3 fish per group). **(A–D)** Bar chart of the log-transformed LDA score of bacterial taxa associated with 4 dpi (red) and 28 dpi (red) *versus* control (green) showing the presence of top 20 species in nose **(A, B)** and pharynx **(C, D)** of common carp by LEfSe (*P* < 0.05). **(E–H)** Relative abundance of *Pseudomonadales* **(E)**, *Acinetobacter* **(F)**, *Acidifaciens* **(G)**, and *Sphingobacteriaceae* **(H)** in the nose of common carp in control and SVCV-infected groups of 4 and 28 dpi. **(I–L)** Relative abundance of *Burkholderiales* **(I)**, *Rhodocyclales* **(J)**, *Acidovorax* **(K)**, and *Acidovorax* **(L)** in the pharynx of common carp in control and SVCV-infected groups of 4 and 28 dpi. *p < 0.05, **p < 0.01, ***p < 0.001 (unpaired Student’s t test).

LDA effect size (LEfSe) analysis was used to further explore the changes of microbial composition in the nose and pharynx of common carp after SVCV infection. Here, we identified significant changes in the nose. Specifically, the abundance of *Pseudomonadales*, *Moraxellaceae*, and *Acinetobacter* remarkably increased (~4.2-fold, ~4.1-fold, and ~2.6-fold, respectively) in the nose at 4 dpi, while it decreased at 28 dpi (~3.9-fold, ~3.8-fold, and ~3.9-fold, respectively). In contrast, *Enterococcaceae* decreased more than ~3.4-fold at 4 dpi but ascended at 28 dpi (**Figures 6A, B** **)**. Although the microbiota with different abundance in the pharyngeal mucosa was not significantly different from that in the nasal mucosa during infection, we clearly detected that the microbial diversity in the pharynx changed significantly at 28 dpi. For example, the abundance of *Johnsonii*, *Helveticus*, *Thermaceae*, *Spirochaetaceae*, and *Megasphaera* increased more than ~3.4-fold at 28 dpi, which was not detected at 4 dpi (**Figures 6C, D** **)**. Additionally, we performed scatter diagrams to illustrate the changes in dominant bacteria both in the nose and pharynx (**Figures 6E–L** and **Supplementary Figure 5**). Interestingly, the abundance of pathogenic bacteria *Pseudomonadales* and *Acinetobacter* was significantly increased, and the symbiotic *Acidifaciens* and *Sphingobacteriaceae* were decreased in the nose at 4 dpi, indicating that a secondary bacterial infection might occur after viral infection (**Figures 6E–H** **)**. However, we observed that the abundance of symbiotic bacteria *Rhodocyclales* and *Caulobacte*r decreased significantly, along with pathogenic bacteria *Burkholderiales* and *Acidovorax* reduced in the pharynx at 4 dpi (**Figures 6I–L** **)**. Importantly, the dominant bacteria changes differed in the nose and pharynx, which implies that different microorganisms inhabit different geographical structures. Combined, the microbiome results indicate that the invasion of the virus disrupts the balance of the microbial ecosystem on the mucosal surface, and over time, a new balance was reestablished through a combination of the immune response and microbes.

## Discussion

Synergistic and competitive interactions between host immunity and microbiota emerged in both primitive and modern vertebrates to maintain mucosal tissue surface homeostasis and respond to disease (2, 4). The upper respiratory tract of mammals is populated with a vast range of commensals and potentially pathogenic bacteria, which occupy different ecological niches and form a complex microbial community. Studies have shown that viral invasion disrupts the balance of the respiratory tract surface microbiota, leaving the host vulnerable to respiratory diseases (14, 31). Although the nose and pharynx of teleost have recently been identified as mucosal immune sites with immune responses and microbiota (8, 9), the links between the immune process and microbial dysbiosis after SVCV infection are poorly understood. In this study, comparative transcriptome and microbial analyses were performed in the nose and pharynx of SVCV-infected common carp to characterize the differences between these two distinct tissues, which are separated due to the lack of a choana. Furthermore, our study also revealed a possible virus-induced immune-microbial interaction in the nose and pharynx of teleost.

To induce a strong immune response in the nose and pharynx and explore the dynamics between immune responses and microbiota, we constructed an infection model of common carp *via* intraperitoneal SVCV injection. The common carp began to manifest significant clinical symptoms 3 days after infection, including exophthalmos, pale gills, loss of scales, and caudal fin bleeding, which were similar to those reported in the literature (21). Interestingly, the virus was also detected in the nose and pharynx of infected common carp, which could invade EPC cells and make them appear obvious CPE. Moreover, it is worth noting that viral load changes in the nose and pharynx were similar. Specifically, viral loads increased first and then decreased over time, reaching a peak at 4 dpi and returning to normal at 28 dpi. These results were consistent with those in the mammalian upper respiratory tract during influenza A virus (IAV) infection, with IAV titers reaching maximum levels at 3 to 5 dpi and recovering at 7 dpi (32), which also suggested that innate immunity and the ability to clear viruses were developed not only in mammals but also in fish (33). Here, we focused on the structural and pathological changes in the nose and pharynx tissues of common carp after SVCV infection, as previous studies had reported that viral infection could cause olfactory organ and pharyngeal epithelium damage (10, 34). Similarly, the olfactory organ of SVCV-infected common carp showed a significant enlargement of the LP, which may be caused by increased vascularization and infiltration of neutrophils, macrophages, and lymphocytes in the LP (34). Moreover, obvious hyperplasia of mucus cells was observed both in the olfactory and pharyngeal epithelium of infected fish. The emergence of these phenomena is consistent with previous studies in which the increased amounts of mucus resulted in the secretion of specialized substances (e.g., peroxidase, lysozyme, immunoglobulins, and c-reactive proteins) in the mucus coupled with a series of immune responses, eventually thinning the epithelium and destroying the mucosal barrier (10). Overall, our results demonstrated that the virus successfully invades nasal and pharyngeal mucosal tissues after intraperitoneal injection and replicated in large numbers at the site of entry, leading to tissue damage.

Upon viral infection, the immune system becomes activated to defend the host and eliminate the virus. In mammals, mucosal homeostasis was maintained by the maturation of mucosa-associated lymphoid tissue and the regular exchange of dynamic interaction signals of the immune system (35–37). However, teleost are lower vertebrates and therefore lack mucosa-associated lymphoid tissues in mucosal sites and instead possess a wide range of immune cells and molecules (5). In our study, we detected the changes in immune-related gene expression levels in the nose and pharynx at different time points after SVCV infection *via* qRT-PCR. Antiviral genes were significantly upregulated at 4 and 7 dpi, including pattern recognition receptors (IRF3 and TLR7), antiviral agent interferon-related genes (IFNa1, ISG15), myxovirus resistance protein (MX1), and an RNA editing enzyme (ADAR). As key components of cytokines, which are involved in the intercellular regulation of the immune system and mediate inflammatory responses, interleukins also play a fundamental role in the innate immune response against viruses (12). Our study detected a high expression of interleukins including IL1β, IL6, IL2, and IL8 in the nose and pharynx after SVCV infection. These observations were consistent with previous results and further suggested that innate immunity represented the first line of defense against pathogens, which was effective at developing induced responses and creating inflammatory conditions after initial pathogen exposure (38). In addition to innate immune genes, immunoglobulin genes (IgM, IgZ1, IgZ2, and IgD), the main representatives of adaptive immunity, were also significantly upregulated at 14 dpi, especially in the pharynx. Interestingly, we found significant increases in the mRNA expression levels of hepcidin in the nose and pharynx after SVCV infection. This short peptide is a broad-spectrum antimicrobial agent that possesses both bactericidal and fungicidal properties and also triggers specific host defense responses (39). More importantly, consistent with the viral load and pathological change analyses in the nose and pharynx, the expression levels of immune-related genes gradually recovered to normal at 28 dpi. We also performed RNA-seq analyses to further explore the immune reaction kinetics of the nose and pharynx at 4 and 28 dpi. PCA and volcano plot analysis indicated that the changes in gene expression in both the nose and pharynx were similar at 4 and 28 dpi, with the 28 dpi group being closer to the control group and distant from the 4 dpi group. Similarly, 20 immune-related genes including antiviral, inflammatory, antigen presentation, and antibacteria genes were characterized *via* transcriptome analysis. Consistent with the qRT-PCR results, the expression level of antiviral genes was most significantly upregulated at 4 dpi. In addition to interleukins, we also found other upregulated proinflammatory cytokines, including CXCL, PPAR, DUSP, and CYP. As mentioned above, the presentation of MHC/antigen complexes alongside costimulatory molecules and proinflammatory cytokines could induce an appropriate immune response (40). Studies have also shown that HSP90 could bind to HSC70 and participate in antigen presentation of DC cells as a chaperone molecule (41). In our study, MHC class I was highly expressed at 4 dpi, whereas MHC class II was more highly expressed at 28 dpi. We also found that HSP90 in the nose was upregulated at 4 and 28 dpi, whereas HSP90 in the pharynx was only upregulated at 28 dpi. C3b (a complement component) and FII (a coagulation factor) were upregulated at 4 dpi and are also known effector sites of antigen presentation. Notably, the expression level of antibacterial genes also increased significantly after SVCV infection, second only to that of antiviral genes. Additionally, the expression of antibacterial genes in the nose was higher than that in the pharynx. We believe that this might be due to the disruption of microbial homeostasis on the mucosal surface and mucosal tissue damage caused by virus invasion, which allowed opportunistic pathogens to invade the tissue, resulting in secondary bacterial infection (31).

KEGG pathway enrichment analyses were performed based on the DEGs in the nose and pharynx at 4 and 28 dpi to further explore the changes in the major biological processes involved in SVCV infection. At 4 dpi, the top 10 pathways enriched in the nose and pharynx were all involved in the host immune response, the most representative of which were pattern recognition receptor (PRR) pathways including the RLR signaling pathway, the TLR signaling pathway, and the NLR signaling pathway. PRRs are known to mediate the host’s perception of the virus and activate inflammatory responses to defend the host and eliminate pathogens. Interestingly, this PRR surveillance system is well conserved across all vertebrates (42–44). Three principal members of RIG-I-like receptors (RIG-I, MDA5, and LGP2) were activated after SVCV infection, after which PAMPs from the SVCV specifically bound to RIG-I and MDA5, which contained an RNA helicase domain with an ATP binding site and two caspase activation and recruitment domains (CARDs), thus triggering downstream inflammatory responses (44). Unlike the RLR system, the Toll-like receptors TLR3, TLR7/8, and TLR9 were mainly located in the inner membrane of cells, including the endoplasmic reticulum, endosomes, and lysosomes, all of which were also involved in SVCV recognition and transmission of signals to transcriptional regulatory factors IRF3 and IRF7 to induce inflammation. To our surprise, the pathways involved in the recognition of PAMPs of bacteria were also activated in the TLR pathways, including the TLR2-PI3K-Akt-NF-κB signaling pathway and the TLR4-mediated pathway, which respond to the membrane components of gram-positive or gram-negative bacteria, respectively (45, 46). In the NLR signaling pathway, the bacterial surface ligand PGN was sensed and drove the activation of NF-κB and MAPK. Additionally, guanylate-binding proteins (GBPs) were induced to assemble on the surface of cytosolic bacteria and gram-negative bacteria, after which they signaled caspase1 to induce the production of proinflammatory cytokines. These results further confirmed that SVCV infection altered the susceptibility of the host to pathogenic bacteria. As the level of immune gene expression gradually decreased, increasingly more genes in the nose and pharynx tended to be involved in maintaining the morphology and function of tissues and cells at 28 dpi, such as the ECM–receptor interaction pathway and the focal adhesion pathway.

Based on the expression of antibacterial genes and the activation of bacterial recognition pathways, 16S rRNA sequencing was performed to identify changes in the microbiota composition of the surface of nasal and pharyngeal mucosal tissues at 4 and 28 dpi. Notably, the diversity of the microbiota composition on the mucosal surface of the nose and pharynx at the peak of SVCV infection (4 dpi) was significantly different from that of the control group and the 28 dpi group. Although there was some overlap between the control group and the 28 dpi group, the microbial composition was not identical, indicating that a new homeostasis was formed. Consistent with previous studies on rainbow trout, *Proteobacteria*, *Firmicutes*, and *Bacteroidetes* were among the most abundant microorganisms on the nasal and pharyngeal mucosal surface of common carp, which jointly accounted for 80% of the total microbiota (10, 15). Interestingly, the changes in *Proteobacteria* abundance varied in the nose and pharynx, increasing in the nose and decreasing in the pharynx at 4 dpi. This phenomenon has been identified as a characteristic of microbial disorder and disease in mammals (47). As mentioned above, previous studies on mammals have proposed that some *Clostridiales* such as *Clostridium difficile* can cause intestinal inflammation, whereas others are beneficial taxa that use human milk oligosaccharides (HMOs) to ferment dietary nondigestible carbohydrates into short-chain fatty acids (SCFAs) (48, 49). In our study, the abundance of *Clostridium* in the nose and pharynx increased after SVCV infection compared to the control group; however, the specific function of this bacterial genus in teleost remains unknown. At the genus level, the top 20 bacteria in the nose and pharynx were clustered in the control group and the 28 dpi group, and were separated from the 4 dpi group. Moreover, an increased abundance of *Chryseobacterium*, *Acinetobacter*, and *Streptococcus* was observed in the nose and pharynx, all of which have been associated with human upper respiratory tract infections (50–52). Our findings suggest that SVCV infection disrupts microbial homeostasis on mucosal surfaces and affects partial bacterial colonization. Consistent with these findings, we identified the top 20 bacteria that underwent significant changes in each group in the nose and pharynx. In the nose, the bacteria with significant differences in the control group were mainly beneficial, whereas the bacteria with significant differences in the 28 dpi group were mainly symbiotic. In contrast, in the pharyngeal mucosa, we found that the control group was dominated by symbiotic bacteria, whereas the 28 dpi group had both probiotic and symbiotic bacteria. We suspect that the main reasons for this result are the isolation and environmental differences of the nose and pharynx of teleost. Furthermore, the abundance of opportunistic or pathogenic bacteria such as *Pseudomonadales*, *Acinetobacter*, and *Myxococcales* in the nose and *Streptococcus* and *Alactolyticus* in the pharynx increased significantly at 4 dpi. *Pseudomonas aeruginosa*, a representative pathogenic bacterium belonging to the *Pseudomonadales*, was found to upregulate proinflammatory cytokines, resulting in neutrophil infiltration and a decrease in natural killer (NK) cells and dendritic cells (DCs) at the wound site (53). Studies have also shown that *Pseudomonadales* proliferated and colonized the surface of the upper respiratory tract after IAV infection, thus coinfecting the host alongside the virus (32). Similarly, *Acinetobacter* can cause a wide range of infections, including respiratory infections, endocarditis, and meningitis, which also induce the activation of the Toll-like receptor signaling pathway and the NOD-like receptor signaling pathway to trigger a strong innate immune response (51). In contrast, we found a significant decrease in *Burkholderiales* (i.e., an opportunistic pathogen) abundance in the pharynx at 4 dpi. This result was consistent with the expression of antibacterial genes in the nose and pharynx. Based on this result, we speculated that the incidence of secondary pathogenic bacteria infection in the nose would be higher after SVCV infection.

Our findings indicated that the SVCV successfully invaded the nasal and pharyngeal mucosa tissues after intraperitoneal injection, thus causing histopathological changes and upregulation of immune-related genes. The microflora of the nasal and pharyngeal mucosa surfaces was studied based on the expression of antibacterial and AMP genes and the activation of PRR pathways. As expected, our study found that there was an increase in pathogenic bacteria after SVCV infection. Moreover, although the gene expression and pathway enrichment in the nose and pharynx are similar in response to the same pathogen, the immune response (particularly antibacterial genes) is stronger in the nose. Furthermore, the increase in pathogenic bacteria in the nose was higher than in the pharynx, suggesting that the nasal mucosa can serve as a better immune effector site and provides a reliable basis for nasal vaccination. In conclusion, our results indicated that SVCV infection causes concurrent immune responses and changes in the microbial composition of mucosal surfaces, after which a new homeostasis was reached in the mucosal tissue over time under the combined influence of immune responses and microbiota.

## Data Availability Statement

The datasets presented in this study can be found in online repositories. The names of the repository/repositories and accession number(s) can be found in the article/**Supplementary Material**.

## Ethics Statement

The animal study was reviewed and approved by the Animal Experiment Committee of Huazhong Agricultural University.

## Author Contributions

Z-BW performed most of the experiments and wrote the manuscript. Z-BW and L-GD analyzed the data. K-FM, SW, G-KH, XZ, R-HS and Y-YY helped with most of the experiments. WJ and ZX designed the experiments and revised the manuscript. All authors contributed to the article and approved the submitted version.

## Funding

This work was supported by grants from the National Natural Science Foundation of China (U1905204, 32073001, and 31873045) and a grant from the Key Laboratory of Sichuan Province for Fishes Conservation and Utilization in the Upper Reaches of the Yangtze River, Neijiang Normal University (NJTCSC01).

## Conflict of Interest

The authors declare that the research was conducted in the absence of any commercial or financial relationships that could be construed as a potential conflict of interest.

## Publisher’s Note

All claims expressed in this article are solely those of the authors and do not necessarily represent those of their affiliated organizations, or those of the publisher, the editors and the reviewers. Any product that may be evaluated in this article, or claim that may be made by its manufacturer, is not guaranteed or endorsed by the publisher.
