# Supplementary material for: Dynamic Interaction Between Mucosal Immunity and Microbiota Drives Nose and Pharynx Homeostasis of Common Carp (Cyprinus carpio) After SVCV Infection
Source: Front Immunol. 2021 Nov 4;12:769775. doi: 10.3389/fimmu.2021.769775 (PMC8601392; doi:10.3389/fimmu.2021.769775)
Supplement: Supplementary file 1 [file DataSheet_1.docx]

**Dynamic interaction between mucosal immunity and microbiota drives nose and pharynx homeostasis of common carp (*Cyprinus carpio*) after SVCV infection**

Zheng-Ben Wu^1^, Kai-Feng Meng^1^, Li-Guo Ding^1^, Sha Wu^1^, Guang-Kun Han^1^, Xue Zhai^1^, Ru-Han Sun^1^, Yong-yao Yu^1^, Wei Ji^1*^, Zhen Xu^2,1*^

^1^Department of Aquatic Animal Medicine, College of Fisheries, Huazhong Agricultural University, Wuhan, Hubei 430070, China

^2^State Key Laboratory of Freshwater Ecology and Biotechnology, Institute of Hydrobiology, Chinese Academy of Sciences, Wuhan, Hubei, 430072, China

^*^Corresponding Author: [zhenxu@ihb.ac.cn](mailto:zhenxu@ihb.ac.cn); wei-ji@mail.hzau.edu.cn.

**Supplementary materials**


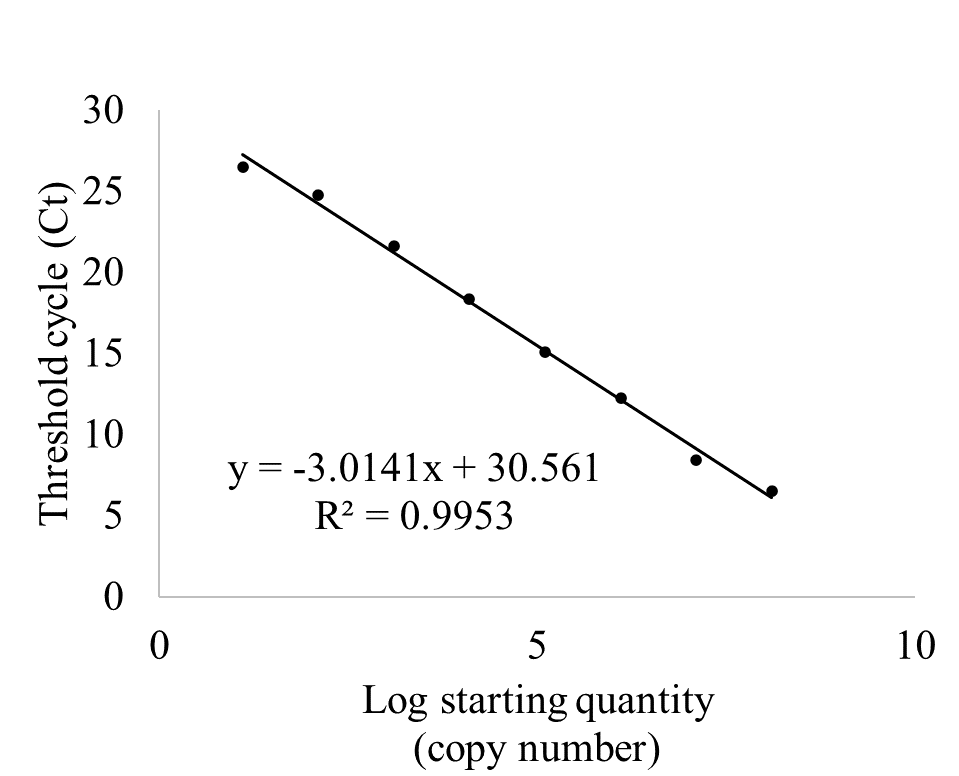


**FIGURE S1** | Standard curve for SVCV load used in this study.

**FIGURE S2** | The width of the OE of nose in control fish and SVCV-infected fish at 1, 4, 7, 14, and 28 dpi (n = 6 fish per group).


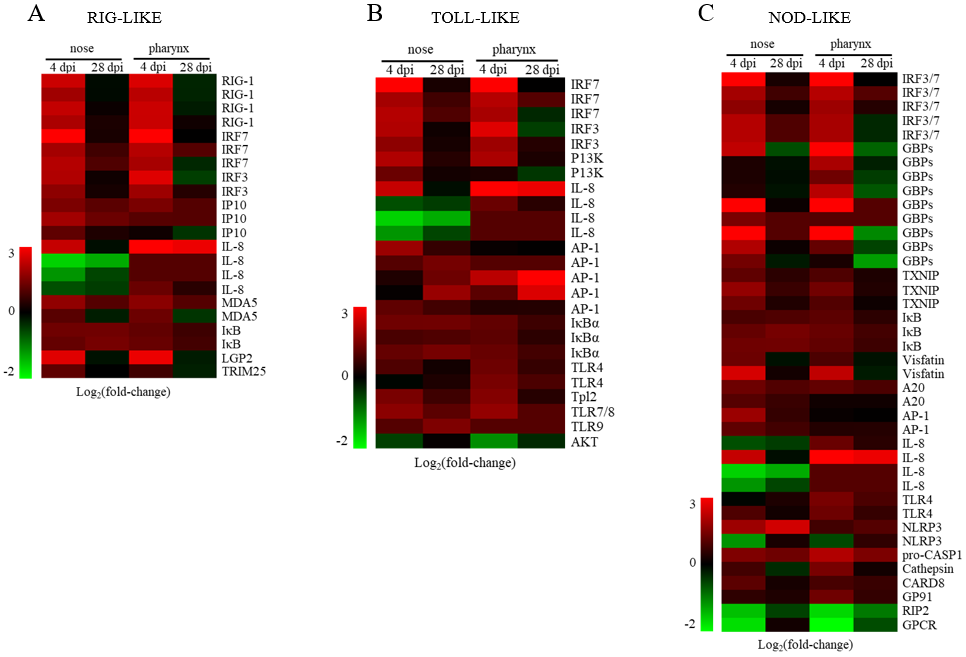


**FIGURE S3** | Heatmap illustrates results from RNA-seq of the mRNA expression levels of genes in RIG-like receptor signaling pathway (A), Toll-like receptor signaling pathway (B), and NOD-like receptor signaling pathway (C) in nose and pharynx of SVCV-infected fish versus control fish measured at 4 and 28 dpi (n = 3 fish per group). Data are expressed as logFC. FC, fold changes.


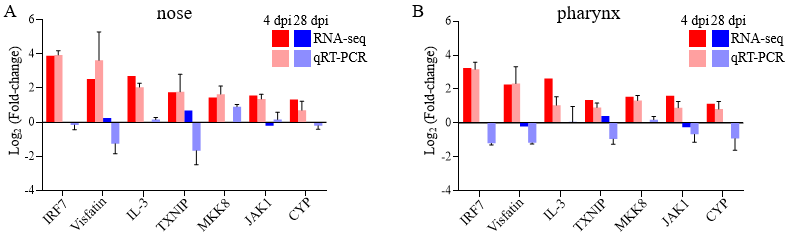
**FIGURE S4** | Transcriptomic differential expressed genes in experimental groups were detected using qRT-PCR to validate RNA-seq. Positive numbers in the Y axis mean up-regulated, while negative values mean down-regulated.


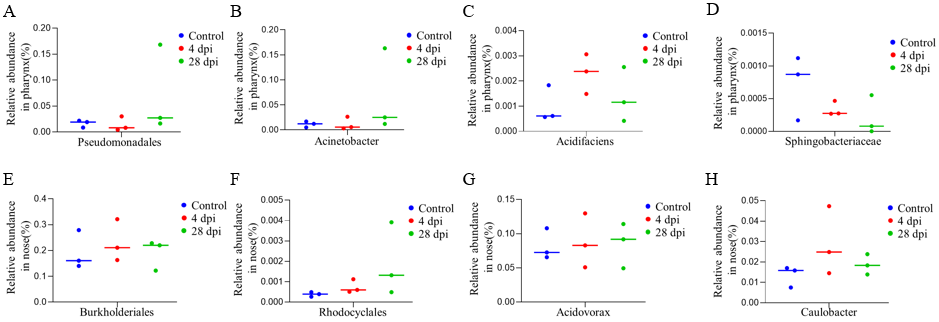
**FIGURE S5** | (A-B) Relative abundance of Pseudomonadales (A), Acinetobacter (B), Acidifaciens (C), and Sphingobacteriaceae (E) in the pharynx of common carp in control and SVCV-infected at days 4 and 28. (I-L) Relative abundance of Burkholderiales (E), Rhodocyclales (F), Acidovorax (G), and Acidovorax (H) in the pharynx of common carp in control and SVCV-infected groups at days 4 and 28.

**TABLE S1 |** Primers used in this study.

| **Gene** | **Full name** | **GenBank**  **accession no.** | **Primer Sequence (5’-3’)** | **Tm**  **(◦C)** | | | **Amplicon**  **Length (bp)** |
| --- | --- | --- | --- | --- | --- | --- | --- |
| **Reference gene** | | | | | |  |  |
| 40S | 40S ribosomal protein | AB_012087.1 | F: CCGTGGGTGACATCGTTACA  R: TCAGGACATTGAACCTCACTGTCT | | 58 | | 69 |
| **Spring viremia of carp virus** | | | | | |  |  |
| SVCV-N | Spring viremia of carp virus | DQ_491000.1 | F: CTCTGCCAAATCACCATACTC  R: GCGGTTTTCTGTATGTGTCTC | | 58 | | 224 |
| **Anti-viral genes** | | | | | |  |  |
| ISG15 | Interferon-stimulated gene 15 | KP_115358.1 | F: AAGCCATATTCAGCGAAGC  R: AACCGTTATCGGCAGACAG | | 58 | | 185 |
| IRF3 | Interferon regulatory factor 3 | JQ_478481.1 | F: GGAGACCACTCTGTTTGGAAG  R: CGGCATCGTTCTTGTTGTC | | 58 | | 88 |
| PKR | Protein kinase R | EX_880666.1 | F: CCAACATCGTCCGCTACTACTC  R: GCGTGTCTCCCTCACAAAG | | 58 | | 147 |
| MX1 | Myxovirus resistance 1 | KP_115357.1 | F: GGCTGGAGCAGGTGTTGGTATC  R: TCCACCAGGTCCGGCTTTGTTAA | | 58 | | 255 |
| ADAR | Adenosine deaminase that acts on RNA | EC_392392.1 | F: GCAGGACGAGGTGTCAGAG  R: GGCAAAGGGAGCATAACTTC | | 58 | | 200 |
| TLR7 | Toll-like receptor 7 | AB_553573.1 | F: AAAGTCTTCGTCAGCACCAGCG  R: CTCTCCGAAGCACAGGTAGATGGT | | 58 | | 104 |
| IFN a1 | Interferon a1 | AB_376666.1 | F: CAGAGTCAATGCTCCGCTTG  R: CAAGAAACCTCACCTGGTCCTC | | 58 | | 178 |
| **AMPS genes** | | | | | |  |  |
| Hepcidin | Hepcidin | KC_795559.1 | F: GCATGCGTCTGCATCCTCC  R: CTGGTTCTCCTGTGGTGCTT | | 58 | | 96 |
| NKL2 | Natural killer lysin 2 | KX_034213.1 | F: GTCCTGATCACCCTGCTGAT  R: AGCACTTTCCAGGGAGTTGT | | 58 | | 130 |
| APOA1 | Apolipoprotein A-Ⅰ | AJ_308993.1 | F: CCATCTCCGCCTCCTTTC  R: ATGTGTTAGTGTGTGTGTGCTTC | | 58 | | 123 |
| APOA14 | Apolipoprotein A-Ⅱ | JQ_038773.1 | F: CACCAACAGGAGGACAAGCCAAAG  R: GCCATAAGCACCAAGAAGAGCCAAG | | 58 | | 149 |
| **Igs genes** | | | | | |  |  |
| IgM | Immunoglobulin M | AB_004105.1 | F: TAGTGCCTCCCTCCCTTGA  R: AGTGCCGTTGCTCCATTCT | | 58 | | 200 |
| IgZ1 | Immunoglobulin Z1 | AB_598367.1 | F: CCAAGAAGGCAACATCATCA  R: AGTGAGGTTCCTGGGGTAGA | | 58 | | 211 |
| IgZ2 | Immunoglobulin Z2 | AB_598368.1 | F: CCATGTGCGTATCAGTAAAAGT  R: CTCTAGTGAGGTGCCTTCAGA | | 58 | | 276 |
| pIgR | Polymeric immunoglobulin receptor | GU_338410.1 | F: GATGACCCAGAATCACCCCC  R: GTCAGACACCCACATTCCCC | | 58 | | 157 |
| IgD | Immunoglobulin D | AB_774152.1 | F: TTGGTTGTTGGTCAGAGT  R: TTGGATTGTGAACGATGC | | 58 | | 70 |
| **Inflammatory genes** | | | | | |  |  |
| IL1β | Interleukin 1β | AB_010701.1 | F: CAGAGCAACAAACTAAGTGACGAG  R: ACCATCTAACTGGGTACAAGCAAG | | 58 | | 189 |
| IL6 | Interleukin 6 | AY_102632.1 | F: GTTTACACCCACCTGAAGGAGTT  R: GATTTCTAAGATACAGTTCACCCTCAC | | 58 | | 159 |
| IL2 | Interleukin 2 | AF_486820.1 | F: CGAACGGGACGAGAAATGG  R: TGATAAAGAGCTGCTGTGAATG | | 58 | | 152 |
| IL8 | Interleukin 8 | KU_881637.1 | F: GGGTGTAGATCCACGCTGTC  R: AGGGTGCAGTAGGGTCCAGA | | 58 | | 167 |

F: Forward primer; R: Reverse primer.
